# A Detailed Study to Discover the Trade between Left Atrial Blood Flow, Expression of Calcium-Activated Potassium Channels and Valvular Atrial Fibrillation

**DOI:** 10.3390/cells11091383

**Published:** 2022-04-19

**Authors:** Pin Shen, Misbahul Ferdous, Xiaoqi Wang, Guojian Li, Runwei Ma, Xiangbin Pan, Hongming Zhang, Guimin Zhang, Zhiling Luo, Lakshme Kottu, Jiang Lu, Yi Song, Lin Duo, Jianming Xia, Enze Yang, Xiang Cheng, Manning Li, Shaohui Jiang, Yi Sun

**Affiliations:** 1 Department of Cardiovascular Surgery, Fuwai Yunnan Cardiovascular Hospital, Kunming 650102, China; shenpin@kmmu.edu.cn (P.S.); wangxiaoqi6@kmmu.edu.cn (X.W.); marw0102@aliyun.com (R.M.); xiangbin428@hotmail.com (X.P.); luozhl2@126.com (Z.L.); yangrongyi1227@126.com (J.L.); drsongyi@163.com (Y.S.); duolin@hotmail.com (L.D.); kmykdxxwkx@163.com (J.X.); yez19960530@163.com (E.Y.); cx0105cx@163.com (X.C.); lmn15383659477@163.com (M.L.); jiangshaohui95@163.com (S.J.); 2 National Center for Cardiovascular Diseases, Department of Cardiology, Fuwai Hospital, CAMS&PUMC, Beijing 100037, China; drmisbah.cardio@gmail.com; 3 Department of Vascular Surgery, The Second People’s Hospital of Yunnan Province, Kunming 650021, China; liguojian81@126.com; 4 Department of Engineering Mechanics, Kunming University of Science and Technology, Kunming 650500, China; hmzhang8888@126.com; 5 Department of Cardiovascular Surgery, The Second People’s Hospital of Yunnan Province, Kunming 650021, China; zgminpaper@163.com; 6 Department of Experimental Cardiology, Erasmus University Medical Center, 3015 CE Rotterdam, The Netherlands; drlakshme3@gmail.com

**Keywords:** valvular atrial fibrillation, turbulent shear stress, IKCa2.3/3.1, thrombosis, computational model

## Abstract

Background: The present study aimed to explore the correlation between calcium-activated potassium channels, left atrial flow field mechanics, valvular atrial fibrillation (VAF), and thrombosis. The process of transforming mechanical signals into biological signals has been revealed, which offers new insights into the study of VAF. Methods: Computational fluid dynamics simulations use numeric analysis and algorithms to compute flow parameters, including turbulent shear stress (TSS) and wall pressure in the left atrium (LA). Real-time PCR and western blotting were used to detect the mRNA and protein expression of IKCa2.3/3.1, ATK1, and P300 in the left atrial tissue of 90 patients. Results: In the valvular disease group, the TSS and wall ressure in the LA increased, the wall pressure increased in turn in all disease groups, mainly near the mitral valve and the posterior portion of the LA, the increase in TSS was the most significant in each group near the mitral valve, and the middle and lower part of the back of the LA and the mRNA expression and protein expression levels of IKCa2.3/3.1, AKT1, and P300 increased (*p* < 0.05) (*n* = 15). The present study was preliminarily conducted to elucidate whether there might be a certain correlation between IKCa2.3 and LA hemodynamic changes. Conclusions: The TSS and wall pressure changes in the LA are correlated with the upregulation of mRNA and protein expression of IKCa2.3/3.1, AKT1, and P300.

## 1. Introduction

Atrial fibrillation (AF) is a common arrhythmia, and it accounts for approximately 20% of all factors causing ischemic stroke [1]. Valvular atrial fibrillation (VAF) is generally rheumatic valvular disease with atrial fibrillation after cardiac valve replacement, and atrial fibrillation based on mitral valve disease. The issue of ‘Valvular AF’ (VAF) definition is of relevance, here we consider VAF as AF in patients with moderate-severe Mitral stenosis with rheumatic heart disease and post valvular replacement. VAF has a higher incidence of thrombosis [2]. Atrial cardiomyopathy refers to ‘Any complex of structural, architectural, contractile or electrophysiological changes affecting the atria with the potential to produce clinically-relevant manifestations’ [3]. Based on the changes in atrial remodeling caused by atrial fibrillation, we tried to explore this mechanism from the perspective of biomechanical changes. AF is widely treated with drugs that including rhythm control, heart rate control, and anticoagulation therapy, but this may lead to clinical problems, such as ventricular arrhythmia, insufficient anticoagulation, or bleeding [4,5]. TSS plays an essential role in the study of heart valve hydrodynamics. Furthermore, changes in TSS may lead to endothelial dysfunction of cardiovascular disease and thromboembolic complications of cardiovascular prosthesis devices [6]. Endothelial IKCas, such as IKCa2.3 and IKCa3.1, are expressed in vascular systems [7]. IKCas channels are expressed in both atria and ventricles, but more in the atria [8]. Previous studies have shown that IKCa plays a vital role in atrial repolarization [9,10,11], and IKCa channel blockers effectively reduce the occurrence of AF [10]. IKCa2.3 is regulated by shear stress [7], and IKCa3.1 acts on endothelial cells to produce pathophysiological changes. Thus, IKCa is expected to become a new target for the treatment of cardiovascular diseases [12,13]. However, little is known about the effect of disease-related shear stress on the function of ion channels. We hypothesize that the IKCa2.3/3.1 pathway may play a crucial role in AF. The aim of this research was to study the TSS and wall pressure in the LA and to explore the correlation between the changes in flow field mechanics in the LA and IKCa2.3/3.1 channels.

## 2. Methods

### 2.1. Patients

**Figure 1:**
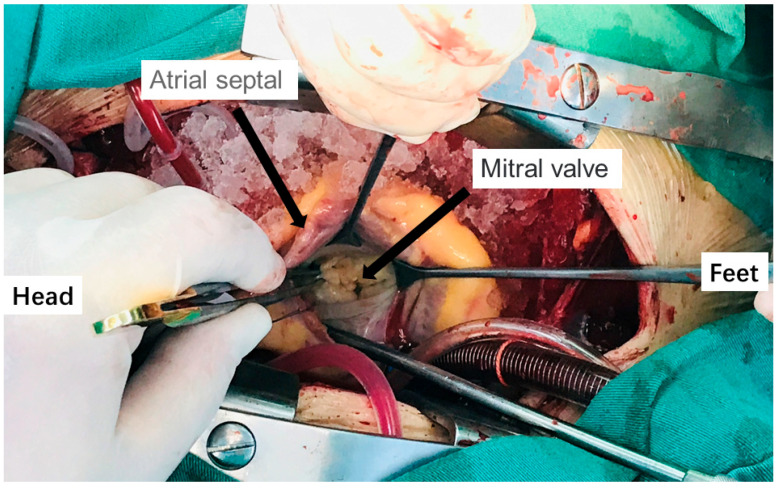
Intraoperative condition of patients undergoing mitral valve replacement.

From April 2016 to October 2019, 75 patients with mitral valve disease, AF, and atrial septal defects were treated in the Department of Cardiac Surgery of the First Affiliated Hospital of Kunming Medical University (Kunming, China) and Fuwai Yunnan Cardiovascular Hospital (Kunming, China), the total number of patients enrolled for study are 80, our of which 75 patients met the inclusion criteria, and 5 patients were dropped out from the study due to acute heart failure and chose conservative treatment. The patients were comprised of 35 males and 40 females with an average age of 55.82 ± 9.28 years and an average weight were 65.29 ± 9.61 kg. The preoperative cardiac function was classified as grade I–III (NYHA standard), including 15 patients with simple mitral regurgitation (MR), 15 patients with simple mitral stenosis (MS), 15 patients with MR+AF, 15 patients with MS+AF, and 15 patients with MS+AF+Thrombus. These clots were in the left atrial appendage. Fifteen patients in the control group had atrial septal defects, ventricular septal defects, and other mild conditions, and they did not have the related hemodynamic changes caused by mitral valve disease, atrial septal defect refers to adult patients who received surgical treatment, but their atrial septal defect was small, and patients without mitral valve disease mainly include patients with aortic dissection, who needed to be hospitalized for emergency surgical treatment. The hemodynamics of the left atrium of these patients have little change and could be used as a control group. In the control group, there were six males and nine females with an average age of 63.00 + 14.68 years and an average weight of 73.32 + 7.15 kg. Information on the clinical data of the subjects is shown in Table 1. All patients underwent surgical treatment. The patient inclusion criteria were as follows: (1) preoperative physical examination, chest X-ray, electrocardiogram, and color Doppler echocardiography all confirmed rheumatic mitral valve disease, and patients had surgical indications and were willing to undergo surgical treatment; (2) patients with persistent atrial fibrillation confirmed by 24-h ambulatory electrocardiogram before operation (MS+AF, MR+AF group); (3) patients with thrombus confirmed by preoperative echocardiography and intraoperative findings (MS+AF+ thrombus group); and (4) all patients provided informed consent and signed the informed consent form, which was authorized by the Ethics Committee of the hospital. Patients with the following conditions were excluded: (1) NYHA IV; (2) malignant arrhythmia; (3) paroxysmal atrial fibrillation; (4) abnormal blood coagulation; (5) cardiomyopathy; (6) diabetes; (7) hyperthyroidism; (8) infection; (9) malignant tumor; and (10) other hereditary diseases or autoimmune diseases. All patients signed a written informed consent form and were reviewed by the Ethics Committee. Tissue from all patients was obtained from the left atrial tissue (atrial septal shown in Figure 1) which separated into two parts during cardiac surgery. One was added to TRIzol reagent for real-time PCR and the other was quickly placed in liquid nitrogen for western blot detection.

### 2.2. CT Scanning and Image Processing

The patients were subjected to a dual-source CT scan with a 2-mm layer thickness. The CT image was processed by MIMICS 19. 0 for 3D reconstruction, so the 3D model of LA could be obtained. The range of the CT image parameters were analyzed in image segmentation adopting 3D threshold segmentation. The threshold ranged from 273 to 502 by repeatedly setting different points, 3D threshold segmentation, and 3D visual display. The elements not required for the present study were manually deleted. The features that needed to be reconstructed were selected to generate the new mask using 3D region expansion. In addition, the unneeded vessels were eliminated by 2D and 3D editing. The components extracted from the CT images included the left superior pulmonary vein (LSPV), right superior pulmonary vein (RSPV), left inferior pulmonary vein (LIPV), right inferior pulmonary vein (RIPV), left atrial appendage (LAA), mitral valve (MV), and LA.

### 2.3. Simulation Model and Calculation Method

First, the CT image was divided into a certain number of meshes, and the segmentation results were then imported into the ANSYS-FLUENT numerical simulation software. ANSYS-FLUENT was utilized to analyze the left atrial flow field and the left atrial inner wall force. After the initial conditions and boundary conditions were determined, the hemodynamic parameters were calculated according to the fluid flow’s basic physical law. The analysis results were imported into the CFD-post calculation software. The following parameters were set: blood vessels were considered impermeable and rigid; blood flow was considered a Newtonian fluid [14]; blood viscosity coefficient of 0.0035 kg/ms [15]; and density of 1.05 × 10^3^ kg/m^3^ at normal body temperature (37 °C) [16].

Our previous study used Doppler ultrasound to construct a calculation method of left ventricular shear stress [17]. We can precisely detect flow field changes and shear stress in the heart by analyzing the data with such a computer [6]. The diastolic inlet and exit velocity spectrum images of the patient’s LA were obtained using Doppler ultrasound before operation. To compare the results, we applied uniform computational fluid dynamics to apply the same boundary and physiological flow conditions to each group.

The calculation type is the transient calculation of unsteady flow. Assuming that gravity is not included, blood flow follows the law of conservation of mass and momentum. The governing equations used in CFD are continuous equations and Navier–Stokes equations for three-dimensional unsteady flow [18] as follows:

$$\rho \frac{DV}{Dt}=-▽p+\mu {▽}^{2}V$$

$$\rho \frac{dv}{dt}=pF+▽\cdot p$$

where p is the intensity of pressure (Pa); *V* is the blood flow velocity vector (m/s); and ρ is the density of the blood (kg/m^3^). The above two formulas are both vector formulas. The continuity equation and Navier–Stokes equation were written in a rectangular coordinate system as follows:

$$\frac{\partial u}{\partial x}+\frac{\partial v}{\partial y}+\frac{\partial w}{\partial z}=0$$

$$\rho \frac{du}{dt}=-\frac{\partial p}{\partial x}+\rho X+\mu \Delta u$$

$$\rho \frac{dv}{dt}=-\frac{\partial p}{\partial y}+\rho Y+\mu \Delta v$$

$$\rho \frac{dw}{dt}=-\frac{\partial p}{\partial z}+\rho Z+\mu \Delta w$$

where Δ is the Laplace operator; ρ is the density of the blood (kg/m^3^); p is the intensity of pressure (Pa); *u*, *v* and *w* are the blood velocities in the directions of the X, Y and Z axes, respectively (m/s); *x*, *y*, and *z* are the forces in the directions of the X, Y, and Z axes (N), respectively; and μ is the viscosity coefficient (Pa·s).

### 2.4. Bioassay

Real-time PCR was used to detect the mRNA expression of IKCa2.3, IKCa3.4, AKT1, and P300 in the patients’ left atrial tissue (atrial septum).

During the operation, we collected atrial septum tissue. The specimen was cut up and placed in a homogenizer followed by the addition of 1 mL of TRIzol reagent (TAKARA, Kusatsu, Japan). By grinding it into a paste on ice, the tissue suspension was obtained. After thoroughly mixing, total RNA was extracted. Reverse transcription was performed according to the reverse transcription kit’s instructions for cDNA. According to the manufacturer’s protocol and then prepared for later qRT-PCR (Bio-Rad, CFX96 Real-Time System, Hercules, CA, USA) using SYBR Green PCR Supermixes (Bio-Rad, Hercules, CA, USA). Moreover, the relative expression level of ribonucleic acid was calculated by the 2^−ΔΔCT^ method. The primer sequences are shown in Table 2, The primers conformed to MIQE guidelines [19].

First, we split the heart tissue, extracted total protein, and then quantified the protein by the BCA method (Wuhan Feiyi Technology Co., Ltd. Wuhan, China). We prepared SDS–PAGE gels (Bioyeartech Company, Wuhan, China), took 80 ug protein samples on the sample pores, carried out electrophoretic, and then transferred them to the membrane. We added the corresponding KCa2.3 antibody (Beijing Zhongshan Jinqiao Company, Beijing, China, NO: 130702), KCa3.1 antibody (Abcam, Shanghai, China, NO: GR98357-2), P300 antibody (abcam, China, NO: GR93557-2), AKT1 antibody (Abcam, China, NO: GR235457-2), or GAPDH antibody (Beijing Bo Osen Company, Beijing, China, NO: 29867) according to the instructions, and diluted the corresponding antibody with 1%BSA solution. The dilution times of the antibodies were 1:500, 1:500, 1:200, and 1:500, respectively, in the refrigerator overnight. We washed the membranes with TBST for 10 min three times. After incubating with the second antibody, Goat Anti-Mouse IgG HRP was diluted to 1:2000 (Beijing Zhongshan Jinqiao Company, Beijing, China, NO: 101966), and Goat Anti-rabbit IgG HRP was diluted to 1:1000 (Beijing Zhongshan Jinqiao Company, Beijing, China, NO: 101964). After that, the TBST membrane was washed three times for 10 min each time, and the protein bands were then exposed using ECL reagent and imaged (Thermo Company, Waltham, MA, USA). Protein bands were analyzed by IMAGEJ.

### 2.5. Statistical Analysis

SPSS 17.0 was used for statistical analysis, and the experimental results are expressed as the mean ± standard deviation. The data of each group were compared with the normality test and the variance test. If the variance was uneven, the variable transformation, variance analysis, and LSD-*t* method were compared in pairs. *p* < 0.05 was considered statistically significant, and *p* < 0.01 was considered a substantial difference.

## 3. Results

### 3.1. Detection of Inlet and Outlet Velocity in LA by Doppler Ultrasonography

Compared to the control group, the pulmonary vein velocity decreased in the MS+AF+ thrombus group, MS+AF group, and MR+AF group, and the decrease in the MS+AF+ thrombus group and MS+AF group was the most significant (*p* < 0.05). There was no significant difference when comparing the MS and MR groups to the control group (*p* > 0.05). Compared to the control group, the LAA velocity decreased in all disease groups, and the difference was statistically significant (*p* < 0.05), especially in the MS+AF+ thrombus group and MS+AF group (Table 3).

### 3.2. Distribution Diagram of LA Wall Pressure in Each Group

**Figure 2:**
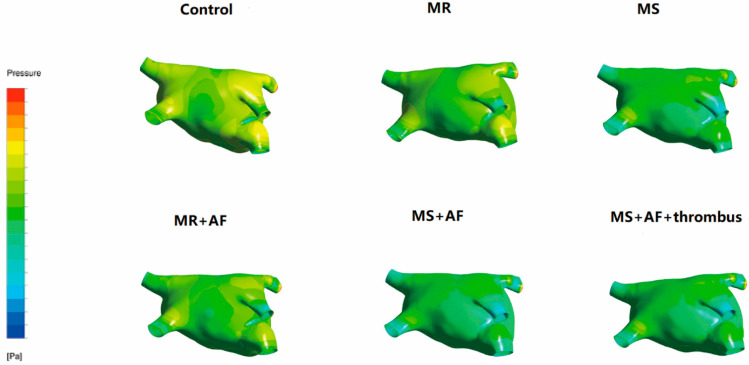
Pressure distribution of LA in the systole stage in front. The color of the legend on the left represents the decreasing wall pressure from top to bottom. (*n* = 15 for each group).

**Figure 3:**
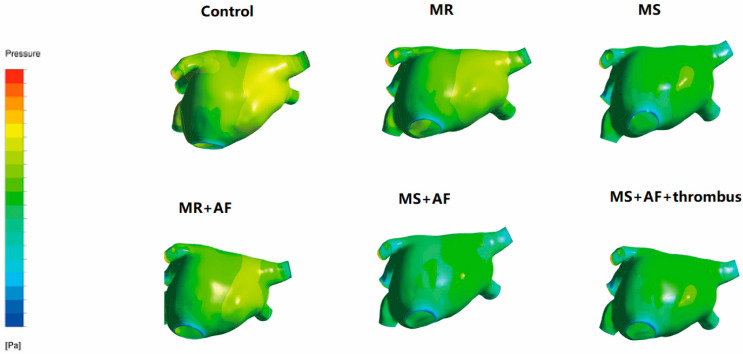
Pressure distribution of LA in the systole stage in back. The color of the legend on the left represents the decreasing wall pressure from top to bottom. (*n* = 15 each group).

**Figure 4:**
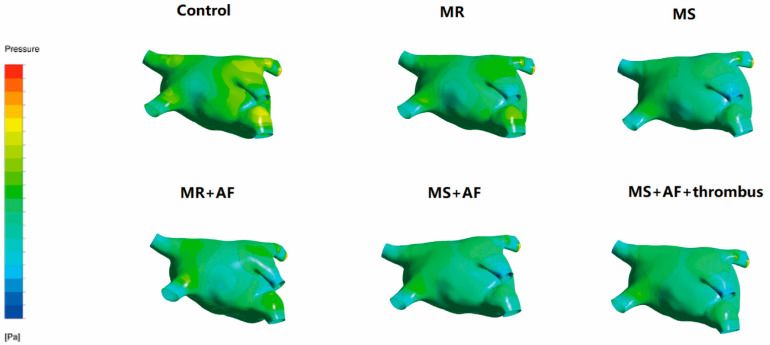
Pressure distribution of LA in the diastole stage in front. The color of the legend on the left represents the decreasing wall pressure from top to bottom. The wall pressure increased in turn in all disease groups, mainly at the entrance of the pulmonary vein, LAA. (*n* = 15 each group).

**Figure 5:**
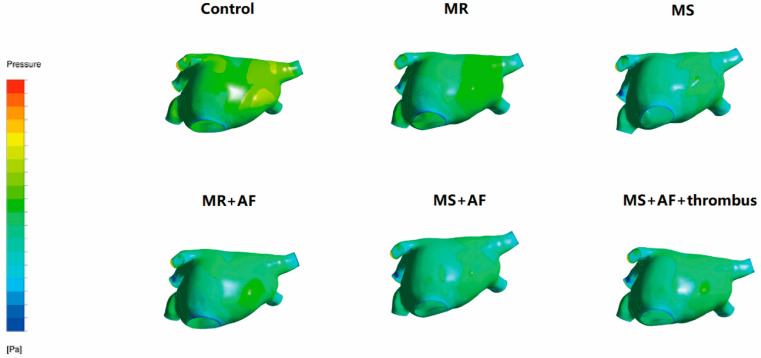
Pressure distribution of LA in the diastole stage in back. The color of the legend on the left represents the decreasing wall pressure from top to bottom. the wall pressure increased in turn in all disease groups, mainly near the mitral valve, and the posterior portion of the LA, the decreasing order of wall pressure was as follows: MS+AF+ thrombus group, MS+AF group, MR+AF group, MS group, MR group, and control group. (*n* = 15 each group).

To obtain better images of the pulmonary vein and LAA, we used CFD-post rotation to rotate the image downward 80° during the process of making the model. Figure 2, Figure 3, Figure 4 and Figure 5 show the pressure distribution in the systolic and diastolic LA of each group. The color of the legend on the left represents the decreasing wall pressure from top to bottom. The results demonstrated that the wall pressure increased significantly in all disease groups in the LA, especially at the pulmonary vein entrance, LAA, near the mitral valve, and the posterior portion of the right atrium. During the systolic and diastolic periods, the decreasing order of wall pressure was as follows: MS+AF+ thrombus group, MS+AF group, MR+AF group, MS group, MR group, and control group. Due to the mitral valve lesion, the LA was enlarged and the congestion in the LA was significantly increased. When the mitral valve was opened during diastole, the release of pressure in the LA was blocked, resulting in high pressure in the left atrial inner wall. When complicated with AF, the LA lost its contractile function, and the release of tension in the LA was further blocked, increasing the pressure of the left atrial inner wall, which was consistent with the increase of TSS.

### 3.3. Distribution Map of TSS in Each Group in LA

**Figure 6:**
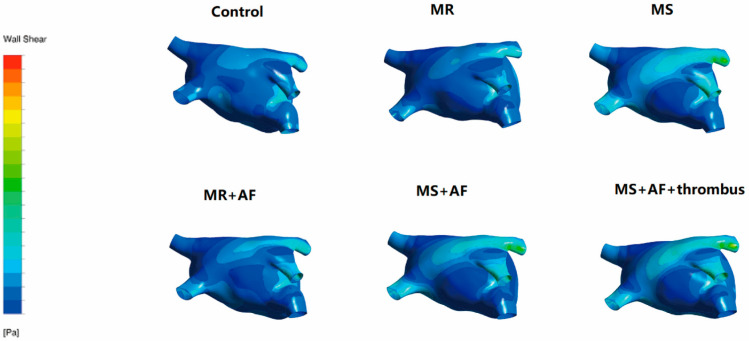
TSS distribution of LA in the systole stage in front. The color of the legend on the left side of the figure represents the decrease in TSS from top to bottom. (*n* = 15 each group).

**Figure 7:**
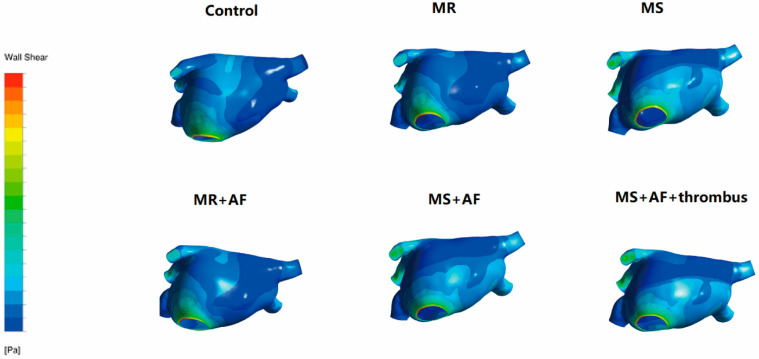
TSS distribution of LA in the systole stage in back. The color of the legend on the left side of the figure represents the decrease in TSS from top to bottom. (*n* = 15 each group).

**Figure 8:**
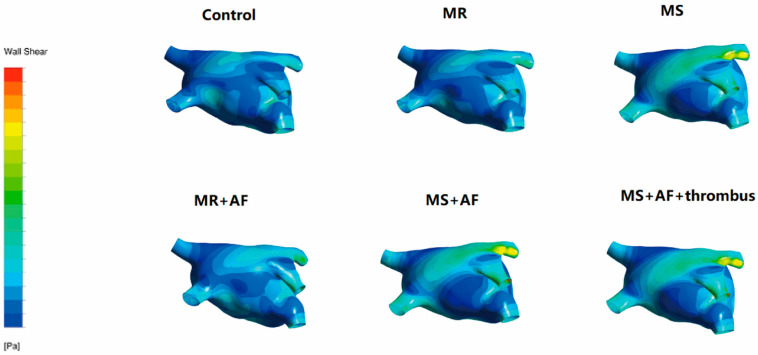
TSS distribution of LA in the diastole stage in front. The color of the legend on the left side of the figure represents the decrease in TSS from top to bottom. (*n* = 15 each group).

**Figure 9:**
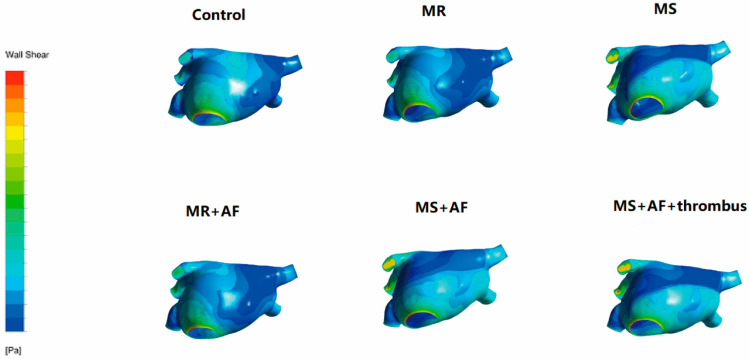
TSS distribution of LA in the diastole stage in back. The color of the legend on the left side of the figure represents the decrease in TSS from top to bottom. The increase in TSS was the most significant in each group near the mitral valve, and the middle and lower part of the back of the LA. The TSS had the following order (from high to low): MS+AF+ thrombus group, MS+AF group, MR+AF group, MS group, MR group, and control group. (*n* = 15 each group).

Figure 6, Figure 7, Figure 8 and Figure 9 show the distribution of TSS in systole and diastole in each group, and the color of the legend on the left side of the figure represents the decrease in TSS from top to bottom. In the LA, the increase in TSS was the most significant in each group at the entrance of the pulmonary vein, the entry of LAA, near the mitral valve, and the middle and lower part of the back of the LA. In the systolic and diastolic phase, the TSS had the following order (from high to low): MS+AF+ thrombus group, MS+AF group, MR+AF group, MS group, MR group, and control group.

The results of LA wall pressure and TSS measurements indicated that in mitral valve disease, especially in VAF, the flow field uniformity of the LA was the worst, and the increase in TSS and wall pressure was the most significant. In addition, these results indicated that the stress site of the LA was consistent with the increase in wall pressure. Mitral valve disease’ was also highly compatible with the development of clinical disease and thrombosis. When heart valve disease occurs, the volume of the LA expands, and atrial congestion increases. Increased intra-atrial pressure, especially in VAF, and thrombosis are common in mitral stenosis with AF and occurred at the orifice of the LAA.

### 3.4. Real-Time PCR

**Figure 10:**
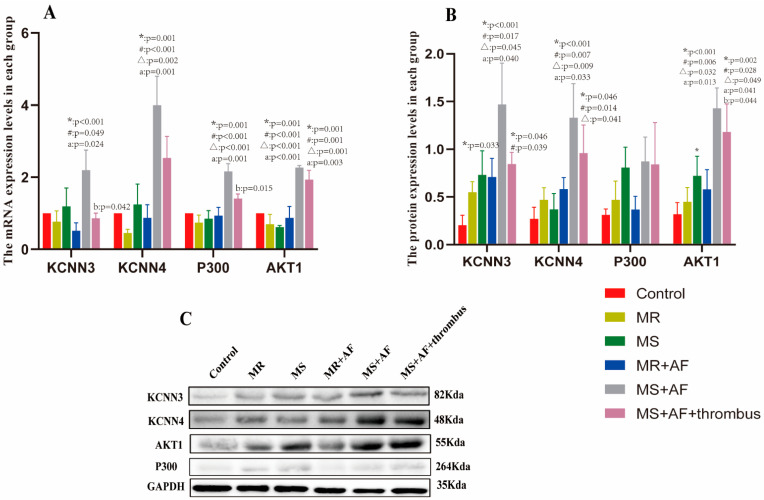
mRNA and protein expression of IKCa2.3(KCNN3), IKCa3.1(KCNN4), AKT1, and P300 in myocardial specimens. (**A**) Mean level of mRNA expression in all patients. (**B**) Mean level of protein expression in all patients. MEAN ± SEM shown as bars. * significant differences from the control group; ^#^ significant differences from the MR group; ^Δ^ significant differences from the MS group; a significant differences from the MR+AF group; b significant differences from the MS+AF group. (**C**) A representative set of western blot.

The KCNN3 and KCNN4 genes encode IKCa2.3 and 3.1, respectively. The mRNA expression levels of IKCa2.3, IKCa3.1, AKT1, and P300 in MS+AF group was significantly differences with the control group (Table 4, Figure 10A). There were significant differences among the MS+AF+ thrombus group, MS+AF group, and control group (*p* < 0.01). by comparing mRNA expression between the MS+AF and MS groups as well as between the MS+AF and MR+AF groups, we found significant difference in IKCa2.3, IKCa3.1, AKT1 and P300 mRNA expression levels. The expressions of IKCa2.3 and P300 mRNA in MS+AF group were higher than those in MS+AF+ thrombus group (*p* < 0.05). Nevertheless, there was no significant difference among the other groups. We compared the mRNA expression of IKCa2.3, 3.1, AKT1 and P300 between the MS and MR groups, and the results showed that there was no significant difference between these groups.

### 3.5. Western Blot

We found that the protein expression of IKCa2.3/3.1, and AKT1 in each group was significantly higher than that in the control group (Table 5, Figure 10B). We selected a representative set of Western blot (Figure 10C). The expression of IKCa2.3/3.1 and AKT1 protein in MS+AF group and MS+AF+ thrombus group was significantly different from those in other groups (*p* < 0.05). The protein expression of IKCa2.3/3.1 and AKT1 in MS+AF group and MS+AF+ thrombus group were significantly higher than those in other groups, and that in MS+AF+ thrombus group was lower than that in MS+AF group. There was no significant difference in the expression of IKCa2.3/3.1 protein between MS group, MR group and MR+AF group. The expression of AKT1 protein in MS group was significantly higher than that in control group (*p* < 0.05). Interestingly, the expression of P300 protein was very low among the groups, and there was no significant difference.

## 4. Discussion

Computational simulation models have been widely used to study fluid-tissue interactions, including fluid velocity, fluid pressure, and heart TSS, in various sections of the mammalian heart [20]. The computational model allows accurate simulations of the fluid values in the heart of mammals and in-depth analysis, and it allows researchers to obtain accurate and reasonable experimental results. By collecting clinical data using ultrasonic Doppler and MRI, as well as establishing the left atrial numerical simulation model, KoizumiR et al. [21] found that when AF occurs, the hemodynamics of the LA significantly change, resulting in blood stasis. The changes in flow field mechanics in the LA may lead to related ion channel expression changes. Takai et al. [22] showed that shear stress in the LA leads to the upregulation of IKCa2.3 and IKCa3.1, which mediates the activation of p300 in an AKT1- and IKCa-dependent manner. With the continuous advancements of computer technology and software, high-performance computers can be used to simulate the changes of the blood flow field of the LA in the diseased state, and by analyzing the changes of the flow field in the LA, the development direction of the disease can be predicted [21,23].

In the present study, we numerically simulated the TSS and wall pressure in the LA. Compared to the control group, the TSS in the atrium at the left and right pulmonary vein entrance showed the greatest increases in the MS+AF+ thrombus, MS+AF, and MR+AF groups. The most apparent change in TSS in the atrium among the groups was at the pulmonary vein entrance. The TSS in each group had the descending: MS+AF+ thrombus group, MS+AF group, MR+AF group, MS group, MR group, and control group. The left atrial wall pressure had the descending: MS+AF+ thrombus group, MS+AF group, MR+AF group, MS group, MR group, and control group. The most significant increase in wall pressure was located at the pulmonary vein entrance and in the LAA, which was consistent with the stress point of TSS in the LA. The above results indicated that the increase in the left atrial wall pressure is highly compatible with the degree of left atrial tissue injury. The atrial damage in the VAF group was more severe than that in the simple valvular disease group. Compared to the simple valvular disease group, the TSS and wall pressure in the LA also increased, and turbulence formation was more pronounced. The abnormality and gradual increase in TSS in the left atrial flow field are fundamental causes of atrial tissue injury and thrombosis. The atrium injury affects the entire cardiac function, resulting in a continuous increase in TSS in the LA, further deterioration of the left atrial flow field’s stability, and progressive damage to the LA, even in the whole heart. These findings were consistent with the progression of VAF observed in the clinic. The impact of atrial cardiomyopathies on the occurrence of atrial fibrillation and atrial arrhythmia, in patients with atrial cardiomyopathy, as a preliminary experiment to explore the biomechanical mechanism, revealed the relationship between cardiac hemodynamic changes and atrial fibrillation. We hope to improve the experimental methods and perfect our research.

Both IKCa2.3 and IKCa3.1 channels are considered potential drug targets for cardio-cerebrovascular disease [24]. In endothelial cells, flow shear stress upregulates the expression of IKCa2.3 and IKCa3.1, and it is mechanically involved in the activation of the CaMKK/AKT/P300 cascade [22]. Blood flow shear stress can cause vascular endothelial cell dysfunction, resulting in IKCa3.1 activation of platelets [25]. The present study used real-time PCR and Western blot to detect IKCa2.3 and IKCa3.1 channel genes and proteins in patients with simple mitral valve disease, patients with VAF, patients with VAF complicated with thrombus formation, and the control group. Compared to the control group, the mRNA and protein expression levels of IKCa2.3 and IKCa3.1, AKT1, and P300 in each group were significantly different from those in the control group. There were significant differences among the MS+AF+ thrombus group, MS+AF group, and control group. There was no significant difference in the mRNA expression and protein expression of IKCa2.3/3.1 among the groups, except there was a difference when comparing the MS and MR groups to the MS+AF groups. These results demonstrated no expression of IKCa2.3 and IKCa3.1 channel genes and proteins in the myocardium of patients with mitral valve disease, but IKCa2.3 and IKCa3.1 gene and protein expression were significantly increased in patients with atrial fibrillation, suggesting that the increased mRNA and protein expression of IKCa2.3 and IKCa3.1 channels may be related to VAF and thrombosis.

P300 is a large histone acetyltransferase that regulates various DNA-binding transcription factors [26]. According to Skikama et al., P300 is necessary to develop a cardiac valvular septal structure and coronary vascular system [27]. The gene expression of P300 was the highest in the MS+AF group, indicating that P300 is involved in VAF formation of VAF and thrombus formation in the LA. P300 in the nucleus affects its expression in each group.

AKT1 is the central node downstream of cellular signals stimulated by growth factors, cytokines, and other cells [28]. The AKT1 signaling pathway mediates insulin-dependent heart growth during postnatal growth and development of Homo sapiens [29]. There was no significant difference in AKT1 gene expression when comparing the MS group to the MR group, and MR+AF group, but there was a significant difference among the other groups. These findings suggested that AKT1 is involved in the development of VAF.

AF patients are in prothrombotic state with slow left atrial blood flow disorder, endocardial damage, increased blood coagulation factor content, enhanced coagulation activity, and hyperfunction platelet activation [30]. The primary conditions for thrombosis are hemodynamic disorder, increased TSS in the LA, and increased compressive stress in the inner wall. The secondary condition for thrombosis is endothelial dysfunction due to the increase in TSS, which leads to endothelial cell dysfunction, resulting in upregulated expression of IKCa2.3, IKCa3.1, AKT1, and P300. Due to the decrease in blood flow velocity in the LA and increased pressure in the LA, coagulation factors, platelets, and other substances are easily deposited in the LA, resulting in thrombus formation. We observed that the expression of IKCa ion channel in patients with thrombus was lower than that in patients with mitral valve disease and atrial fibrillation. Sicne the surface of the left atrium is covered with thrombus, the stress of blood flow on the surface of the atrium is reduced, and there is a lack of stimulation.

In conclusion, we used computer numerical simulation of TSS and wall pressure in the LA, and we also used real-time PCR and western blot analyses to detect the mRNA and protein expression of IKCa2.3, IKCa3.1, AKT1, and P300. The present study demonstrated that the TSS and wall pressure in the LA change during valvular heart disease, resulting in upregulated mRNA and protein expression of IKCa2.3, IKCa3.1, AKT1, and P300. These results suggested that IKCa channels are involved in the pathogenesis of VAF, and can contribute to our understanding of the mechanism of VAF and thrombosis.

At present, due to the complexity of the fluid in the cardiac cavity, we can only construct a transient heart model, assuming that the blood is a Newtonian fluid and the blood vessel wall is rigid. With the development of mechanical software, if the hemodynamic study of the heart can be calculated by the method of fluid-solid coupling and the changes of blood flow in the whole cardiac cycle analyzed, the authenticity of the model will be greatly improved.

## Figures and Tables

**Table 1:** Clinical data of patients.

Group	Gender		Age (Years)	Weight (kg)
	Males	Females		
Control	6	9	63.00 + 14.68	73.32 + 7.15
MR	7	8	55.60 + 12.03	67.65 + 8.42
MS	8	7	52.50 + 16.38	54.78 + 6.21
MR+AF	6	9	58.00 + 10.44	65.31 + 14.22
MS+AF	5	10	59.75 + 4.27	67.84 + 9.75
MS+AF+ thrombus	9	6	53.25 + 3.30	70.87 + 9.46
*p*-value			0.743	0.642

**Table 2:** Primer sequences for IKCa2.3, IKCa3.1, P300, AKT1, and GAPDH.

ID	Forward Primer (5′–3′)	Reverse Primer (5′–3′)
IKCa2.3	GACTTTCACAGACACGGACGG	GCTGCACAGCAAGCTCTTCAC
IKCa3.1	CCAGGCTTCTTGTAGCACTCG	CCCCATCACATTCCTGACCAT
P300	GTAGCAGCAGAGGGCAATGAG	GGCCTTGGCTTAGATGATGAG
AKT1	GGTCGTGGGTCTGGAAAGAGT	GGCAAGGTGATCCTGGTGAAG
GAPDH	GGACCTGACCTGCCGTCTAG	GTAGCCCAGGATGCCCTTGA

**Table 3:** Detection of flow velocity at each inlet and outlet by the Doppler ultrasound. Data are expressed as the mean ± SEM of *n* = 15.

		Control (cm/s) (*n* = 15)	MR (cm/s) (*n* = 15)	MS (cm/s) (*n* = 15)	MR+AF (cm/s) (*n* = 15)	MS+AF (cm/s) (*n* = 15)	MS+AF+Thrombus (cm/s) (*n* = 15)	*p*-Value
Pulmonary vein	LSPV	41.3 ± 20.91	41.3 ± 20.13	47.36 ± 10.34	31.36 ± 4.34	18.01 ± 8.47 *^,#,Δ^	16.61 ± 10.19 *^,#,Δ^	0.031
	LIPV	41.5 ± 17.42	40.9 ± 18.93	47.54 ± 11.36	31.73 ± 6.19	18.27 ± 7.61 *^,#,Δ^	18.24 ± 16.52 *^,#,Δ^	0.003
	RSPV	45.2 ± 19.92	37.8 ± 15.52	48.83 ± 9.69	32.71 ± 8.21	19.69 ± 8.89 *^,#,Δ^	18.19 ± 14.90 *^,#,Δ^	0.024
	RIPV	42.2 ± 17.84	39.1 ± 17.02	46.57 ± 12.26	31.92 ± 6.78	17.69 ± 7.26 *^,#,Δ^	17.26 ± 16.25 *^,#,Δ^	0.001
LAA		56.3 ± 15.12	44 ± 4.84	34.8 ± 10.82	30.7 ± 3.36 *	18.54 ± 17.21 *^,#^	11.8 ± 2.16 *^,#,Δ^	0.019
Mitral orifice		74.67 ± 16.32	78.36 ± 18.14	200.18 ± 14.89 *^,#^	80.17 ± 15.49 ^Δ^	214.67 ± 18.26 *^,#,a^	214.26 ± 16.78 *^,#,a^	0.015

* significant difference from the control group, *p* < 0.05; ^#^ significant difference from the MR group, *p* < 0.05; ^Δ^ significant difference from the MS group, *p* < 0.05; ^a^ significant difference from the MR+AF group, *p* < 0.05.

**Table 4:** mRNA expression levels of IKCa2.3, IKCa3.1, AKT1, and P300 in each group. After the ANOVA for each group (*p* < 0.05), the variance for uneven group was tested using the Welch test, (*p* < 0.05), which means that there were statistical differences in many groups. The data are expressed as the mean ± SEM.

Group	Control (*n* = 15)	MR (*n* = 15)	MS (*n* = 15)	MR+AF (*n* = 15)	MS+AF (*n* = 15)	MS+AF+ Thrombus (*n* = 15)	F	*p* Value
KCNN3	1	0.77 ± 0.51	1.19 ± 0.12	0.51 ± 0.38	2.19 ± 0.96 *^,#,a^	0.86 ± 0.24 ^b^	1.635	0.225
KCNN4	1	0.45 ± 0.17	1.23 ± 0.99	0.87 ± 0.64	4.00 ± 1.38 *^,#,Δ,a^	3.20 ± 1.03	2.309	0.109
P300	1	0.74 ± 0.37	0.85 ± 0.39	0.93 ± 0.41	2.16 ± 0.38 *^,#,Δ,a^	1.40 ± 0.22 ^b^	11.624	0.002
AKT1	1	0.69 ± 0.47	0.61 ± 0.79	0.87 ± 0.56	2.27 ± 0.99 *^,#,Δ,a^	1.93 ± 0.45 *^,#,Δ,a^	7.884	<0.001

* significant differences from the control group, *p* < 0.05; ^#^ significant differences from the MR group, *p* < 0.05; ^Δ^ significant differences from the MS group, *p* < 0.05; ^a^ significant differences from the MR+AF group, *p* < 0.05; ^b^ significant differences from the MS+AF group, *p* < 0.05.

**Table 5:** Protein expression levels of IKCa2.3, IKCa3.1, AKT1, and P300 in each group. After the ANOVA for each group (*p* < 0.05), the variance for uneven group was tested using the Welch test, (*p* < 0.05), which means that there were statistical differences in many groups. Data are expressed as the mean ± SEM.

Group	Control (*n* = 15)	MR (*n* = 15)	MS (*n* = 15)	MR+AF (*n* = 15)	MS+AF (*n* = 15)	MS+AF+ Thrombus (*n* = 15)	F	*p* Value
IKCa2.3	0.23 ± 0.14	0.55 ± 0.19	0.73 ± 0.44 *	0.71 ± 0.34	1.47 ± 0.75 *^,#,Δ,a^	0.84 ± 0.21 *^,#^	10.620	0.042
IKCa3.1	0.27 ± 0.21	0.47 ± 0.22	0.37 ± 0.29	0.58 ± 0.21	1.33 ± 0.62 *^,#,Δ,a^	0.96 ± 0.51 *^,#,Δ^	15.229	<0.001
P300	0.31 ± 0.11	0.47 ± 0.34	0.81 ± 0.37	0.37 ± 0.24	0.87 ± 0.44	0.84 ± 0.76	2.623	1.334
AKT1	0.32 ± 0.21	0.45 ± 0.26	0.73 ± 0.37 *	0.58 ± 0.36	1.43 ± 0.37 *^,#,Δ,a^	1.18 ± 0.51 *^,#,Δ,a,b^	11.711	0.001

* significant differences from the control group, *p* < 0.05; ^#^ significant differences from the MR group, *p* < 0.05; ^Δ^ significant differences from the MS group, *p* < 0.05; ^a^ significant differences from the MR+AF group, *p* < 0.05; ^b^ significant differences from the MS+AF group, *p* < 0.05.

## Data Availability

The datasets used or analyzed during the current study are available from the corresponding author upon reasonable request.
